# Oleoylethanolamide facilitates PPARa and TFEB signaling and attenuates Ab pathology in a mouse model of Alzheimer’s disease

**DOI:** 10.21203/rs.3.rs-2484513/v1

**Published:** 2023-01-20

**Authors:** Michele Comerota, Manasee Gedam, Wen Xiong, Feng Jin, Lisheng Deng, Meng Wang, Jin Wang, Hui Zheng

**Affiliations:** Baylor College of Medicine; Baylor College of Medicine; Baylor College of Medicine; Baylor College of Medicine; Baylor College of Medicine; Janelia; Baylor College of Medicine; Baylor College of Medicine

**Keywords:** Alzheimer’s disease, Microglia, Oleoylethanolamide, PPARα, TFEB

## Abstract

Emerging evidence implicates impaired microglia function and dysregulation of lipid metabolism in Alzheimer’s disease (AD). Oleoylethanolamide (OEA), an endogenous lipid and PPARα agonist, has been shown to promote longevity in *C. elegans* through regulation of lysosome-to-nucleus signaling and cellular metabolism. Using a stable OEA analog, KDS-5104, we found that OEA-PPARα signaling promotes TFEB lysosomal activity independent of mTORC1 and upregulates cell-surface receptor CD36, leading to enhanced microglial Aβ uptake and clearance. These are associated with the suppression of LPS-induced lipid droplet accumulation and inflammasome activation. Chronic treatment of the 5xFAD mice with KDS-5104 restored dysregulated profiles, reduced reactive gliosis and Aβ pathology and rescued cognitive impairments. Together, our study provides support that augmenting OEA-mediated lipid signaling may offer therapeutic benefit against aging and AD through modulating lipid metabolism and microglia phagocytosis and clearance.

## Introduction

Alzheimer’s disease (AD) is the most prevalent age-related neurodegenerative disorder characterized by the accumulation of amyloid beta (Aβ) plaques and neurofibrillary tangles^1^. These pathological hallmarks are accompanied by prominent changes of glial cells in the brain. Genome-wide association studies implicate a contributing role of microglia and its associated pathways such as endocytosis and phagocytosis, lipid metabolism and immune response in the etiology of late-onset AD^2^. Besides the genetic evidence, age is known to be the greatest risk factor. The underlying mechanisms for the age influence are likely complex as aging is known to elicit a multitude of changes at cellular, organelle and system levels. Accordingly, compounds that delay aging and promote longevity may prove efficacious in combating AD.

Oleoylethanolamide (OEA) is an evolutionarily conserved, naturally occurring, lipid that has been shown to extend the lifespan and healthspan of *C. elegans* through lysosome to nucleus signaling and activation of metabolic gene expression^3^. In the mammalian system, OEA is produced in both peripheral tissues and the central nervous system (CNS). While a peripheral effect of OEA in regulating satiety and in promoting lipolysis has been well-established, its role in the CNS is poorly understood. Intriguingly, a recent lipidomics analysis identified OEA and other fatty acid ethanolamide as a lipid class downregulated in the cerebral spinal fluid and plasma of AD patients compared to non-demented controls^4^, raising the possibility that OEA and related lipids may influence AD progression and their circulating levels may serve as useful biomarkers.

The effect of OEA in feeding regulation has been attributed to its binding to the peroxisome proliferator activated receptor alpha (PPARα)^5,6^, a ligand activated nuclear receptor that, upon dimerization with the retinoid X receptor, acts as a potent transcription factor to activate downstream targets involved in energy homeostasis, lipid metabolism, autophagy, and inflammation^7^. PPARα agonists have been shown to provide beneficial effects in AD mouse models by acting on APP processing and Aβ metabolism^8,9^, autophagy and lysosomal pathway^10–12^, and neuroinflammation^13,14^.

PPARα may exert its effect through crosstalk with other transcription factors, among them, the transcription factor EB (TFEB). TFEB is a master regulator of autophagy and lysosome biogenesis that coordinates lysosome nutrient status with mTOR-dependent phosphorylation and nuclear signaling^15^. We and others have reported a potent role of TFEB in mitigating Aβ and tau pathologies through both the autophagy-lysosomal pathway and phagocytosis^16–20^. PPARα and TFEB have an intricate network of regulation in which they share common upstream inducers and downstream targets^7^. Relevant to AD, Raha et al. reported an astrocytic PPARα-TFEB pathway in regulating Aβ clearance^12^. However, the role of OEA in these processes and in AD pathophysiology is not understood.

OEA is a lipid amide that can be hydrolyzed by fatty-acid amide hydrolase (FAAH)^21^. To increase the stability of OEA, Astarita et al. developed an analog, KDS-5104, that is a functional mimetic of OEA and resistant to enzymatic hydrolysis^21^. Using the analog in the current study, we present evidence that OEA/KDS-5104 activates PPARα downstream target CD36 to enhance phagocytosis and TFEB to promote lysosomal clearance, the latter is mTOR-independent. These concerted activities lead to potent microglial Aβ and lipid uptake and clearance and LPS-induced inflammasome activation. Administration of KDS-5104 to the 5xFAD mouse model of AD reversed lipid profile alterations, and microglia and astrocyte reactivity in the brain. These changes were accompanied by attenuated Aβ pathology, improved synaptic integrity and cognitive function in the AD model.

## Results

### Increased PPARα and TFEB signaling by KDS-5104

We first evaluated the expression of PPARα and its downstream targets in postmortem AD brain samples and 5xFAD transgenic mouse model with amyloid beta (Aβ) pathology^22^. We found a decreased PPARα protein levels by Western blotting in AD samples compared to non-dementia controls (Supplementary Fig. 1 a, b). Quantitative real-time PCR (qPCR) analysis showed that the gene expression of *PPARA* and downstream target, cytochrome P450 family 4 subfamily A member 11 (*CYP4A11*), were also reduced in postmortem AD human brains (Supplementary Fig. 1c). While the protein and mRNA levels of PPARα and downstream targets were not significantly changed in the 5xFAD mice (Supplementary Fig. 1d-f), analysis of FACS-isolated microglia identified a reduction of *Ppara* and *Cyp4a11* mRNA levels in 9-month-old 5xFAD mice compared to their wild-type littermates (Supplementary Fig. 1g). These results implicate a reduced PPARα pathway in AD, particularly in microglia.

We thus tested whether OEA could upregulate PPARα activity in microglia given its reported activity in peripheral tissues. Treating the primary microglial cultures with KDS-5104, a stable analog of OEA, showed dose-dependent increases of *Ppara* and its downstream target *Cyp4a11* (Supplementary Fig. 2a).

**Figure 1:**
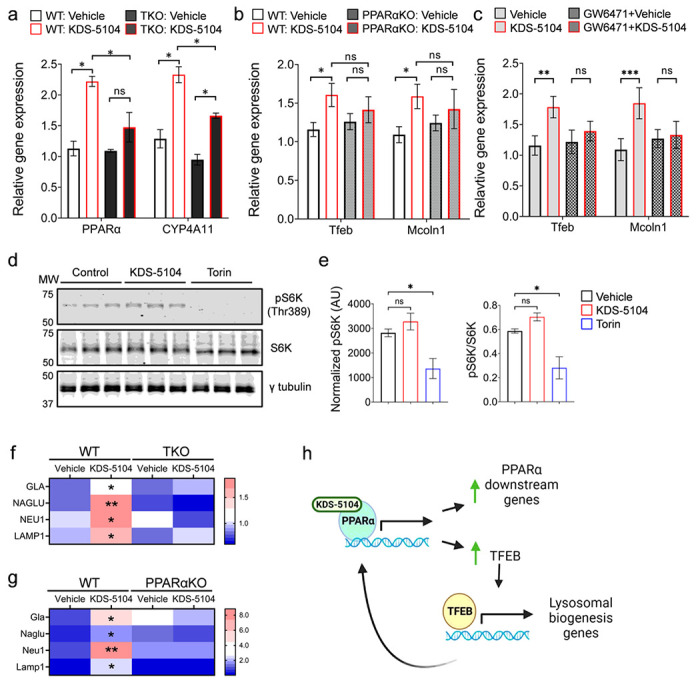
KDS-5104 increases PPARα signaling and TFEB gene expression independently of mTORC1. **a.** qPCR analysis of *PPARA*and *CYP4A11* expression in KDS-5104 treated wild-type (WT) and TKO HeLa cells. **b.** qPCR analysis of *Tfeb* and *Mcoln1* in WT and PPARαKO primary microglial cultures. **c.** qPCR analysis of *Tfeb* and *Mcoln1* in vehicle or KDS-5104 treated BV2 cells with or without pretreatment with PPARα antagonist GW6471. **d.** Western blot analysis of total and phosphorylated S6k (pS6K) levels in primary microglial cultures treated with vehicle (Control), KDS-5104 (10 μM) or Torin (500 nM). g-tubulin was used as a loading control. **e.** Quantification of pS6k expression level normalized to g-tubulin (left) and the ratio of pS6k to total S6k (right). AU: artificial unit. **f.** qPCR analysis of lysosome enzyme genes *GLA, NAGLU, NEU*and *LAMP1*in vehicle or KDS-5104 treated WT and TKO cells. **g.** qPCR analysis of *Gia, Naglu, Neul* and *Lamp1* in vehicle or KDS-5104 treated WT and PPARαKO primary microglia cultures. **h.** Model of KDS-5104 action of PPARα activation and TFEB signaling. For all panels, data are presented as mean ± SEM. ns: non-significant,*p < 0.05, **p < 0.01, ***p < 0.001. One way ANOVA with Tukey’s multiple comparisons tests as the post hoc analysis. Each experiment was repeated 2-3 times each in triplicates.

Since previous studies have reported a crosstalk between PPARα and TFEB, we examined TFEB expression and signaling in these cultures in response to KDS-5104. Interestingly, we detected a similar dose-dependent increases of *Tfeb* gene expression and its downstream target, mucolipin1 (*Mcoln1*) (Supplementary Fig. 2b). To decipher the relationship between PPARα and TFEB, we evaluated the effect of KDS-5104 on PPARα in TFEB/TFE3/MITF triple knockout (TKO) HeLa cells^23^, and conversely, TFEB activity in primary microglia cultured from PPARα knockout (PPARαKO) mice^24^. We found that treating the WT and TKO cells with KDS-5104 resulted in increased *PPARA* and *CYP4A11* expressions, however, the degree of activation was substantially lower in TKO cells (Fig. 1 a), suggesting that OEA/KDS-5104 could act on the PPARα pathway in the absence of TFEB although the maximal activation may require TFEB. In contrary, treating the primary microglial cultures from WT and PPARαKO mice with KDS-5104 showed that, while both *Tfeb* and *Mcoln1* expression were upregulated by KDS-5104, this response was blunted in PPARαKO cultures (Fig. 1b). Similar results were also obtained when BV2 cells were treated with the PPARα antagonist GW6471 (Fig. 1c). Thus, KDS-5104 induces TFEB signaling indirectly through PPARα.

### KDS-5104 activation of TFEB does not require mTORC1

It is well-established that TFEB activity is tightly regulated by mTORC1 through TFEB phosphorylation and nuclear translocation^25,26^. To assess a possible role of mTORC1 in KDS-5104-induced TFEB activation, we measured the levels of the mTORC1 target, phospho-p70 S6 kinase (pS6K) at Thr 389 site^27^, upon treating the HeLa cells with KDS-5104 or mTORC1 inhibitor Torin. A drastic reduction of pS6K were observed when the cells were treated with Torin (Fig. 1d). In contrast, we found no changes of pS6K by KDS-5104 treatment (Fig. 1d and quantified in 1e), suggesting that KDS-5104 does not influence mTORC1 activity. Similarly, KDS-5104 had no effect on Akt activity, which has been reported as mTORC1-independent regulator of TFEB^28^ (Supplementary Fig. 2c, d). These results combined support the notion that KDS-5104 activates TFEB through PPARα-dependent but mTORC1-independent mechanisms.

TFEB is known to be a master regulator of lysosomal biogenesis by activating multiple lysosomal genes^29,30^. We thus aimed to determine if KDS-5104 induces changes of lysosomal genes through PPARα and subsequent TFEB activation. Indeed, qPCR analysis documented an increase in the expression of several TFEB lysosomal targets including alpha-galactosidase A (*GLA*), alpha-N-acetylglucosamindase (*NAGLU*), neuraminidase 1 (*NEU1*), and lysosomal associated membrane protein 1 (*LAMP1*) in KDS-5104 treated WT HeLa cells, which were blunted in TKO cells (Fig. 1f). This is also the case when KDS-5104 were applied to WT primary microglial cultures (Fig. 1g). However, KDS-5104 failed to induce the lysosomal genes in PPARαKO microglia (Fig. 1g), consistent with the idea that PPARα is necessary for KDS-5104 induced TFEB activity.

The results combined support a model whereby KDS-5104 directly acts on PPARα to mediate the expression of its downstream genes and TFEB. TFEB in turn feedback to augment PPARα pathway and also activate its lysosomal targets (Fig. 1h). These effects does not require mTORC1.

### KDS-5104-PPARα signaling promotes lysosomal biogenesis

**Figure 2:**
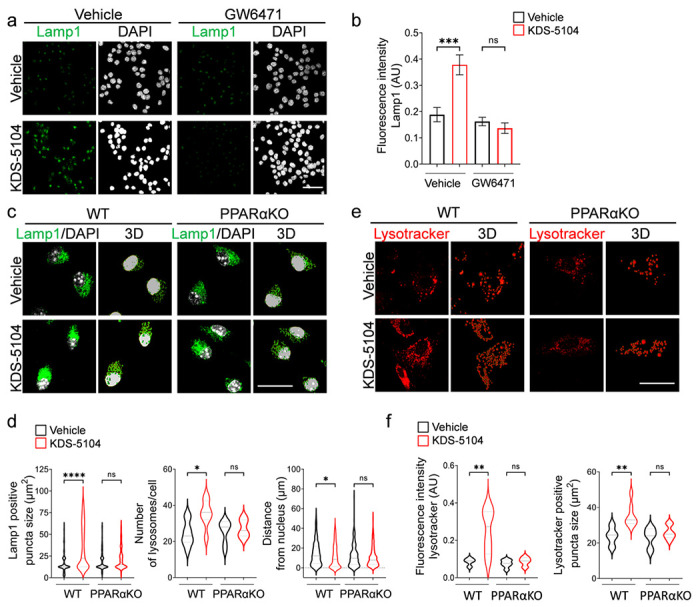
KDS-5104 increases lysosomal biogenesis in a PPARα dependent manner. **a.** Representative Lamp1 immunofluorescence images of vehicle or KDS-5104 treated BV2 cells with or without GW6471 pretreatment. **b.** Quantification of Lamp1 fluorescent intensity. **c.** Representative images of Lamp1 (green) and DAPI (white) staining and corresponding 3D renderings of primary WT and PPARαKO microglia cultures treated with vehicle or 10 μM KDS-5104 for 8 hours. **d.** Quantification of lysosome size, number of lysosomes per cell and distance of lysosomes from the nucleus. **e.** Representative images of lysotracker positive lysosomes and 3D renderings of primary WT and PPARαKO microglial cultures treated with vehicle or 10 μM KDS-5104 for 8 hours. **f.** Quantification of lysotracker fluorescence intensity and number of lysotracker+ lysosomes per cell. AU: artificial unit. Scale bars: 100 μm. For all panels, data are presented as mean ± SEM. ns: non-significant, *p < 0.05, **p < 0.01, ***p < 0.001, ****p < 0.0001. One way ANOVA with Tukey’s multiple comparisons tests as the post hoc analysis. Each experiment was repeated 2-3 times each in triplicates.

We next asked whether increased expression of TFEB lysosomal genes by KDS-5104 is associated with higher lysosomal activity and whether such an effect is PPARα dependent. We treated the BV2 cells with KDS-5104 and performed immunofluorescence staining with an anti-Lamp1 antibody to mark the lysosome (Fig. 2a). KDS-5104 treatment led to higher Lamp1 intensity, indicating increased lysosomal content. Co-treatment with the PPARα antagonist GW6471 abolished the KDS-5104 effects (Fig. 2a, b), consistent with the notion that KDS-5104 promotes lysosomal activity through the PPARα-TFEB axis. To provide additional support, we generated primary microglial cultures from PPARαKO and littermate WT controls and used Imaris imaging software to analyze the properties of lysosomes visualized by lamp1 immunofluorescence (Fig. 2c). In KDS-5104 treated WT microglia, both the lysosomal size and lysosomal number were higher compared to vehicle treated controls (Fig. 2d), indicating an increased lysosomal activity and biogenesis. Further analysis showed that the lysosomes in KDS-5104 treated cells were in closer proximity to the nucleus, further supporting increased lysosomal activity (Fig. 2d). Consistent with the PPARα dependent mechanism, PPARαKO microglia treated with KDS-5104 did not display an increase in lysosome size, number, or altered distance to nucleus compared to vehicle treated PPARαKO microglia (Fig. 2d). Lastly, we utilized a pH sensitive fluorescent dye, lysotracker, to measure lysosomal acidity, which is a common marker of activated lysosomes (Fig. 2e). The KDS-5104 treated WT microglia showed an increase in the lysotracker intensity and lysotracker-positive puncta size, and these effects were abolished in PPARαKO microglia (Fig. 2f). Together, the increase in lysosome size, number, acidity and proximity to nucleus by KDS-5104 treatment in WT but not PPARαKO microglia provide strong support that KDS-5104 promotes lysosomal activity and biogenesis in a PPARα dependent manner.

### KDS-5104 promotes microglial Aβ phagocytosis through PPARα-CD36 axis

**Figure 3:**
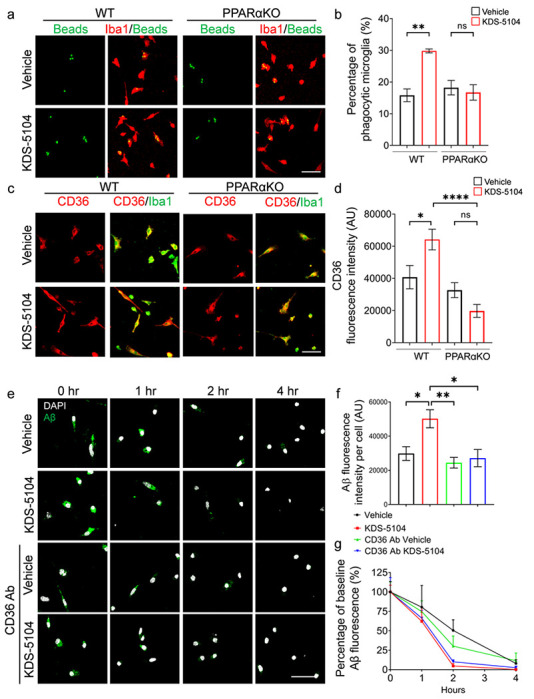
KDS-5104-PPARα promotes phagocytosis and Ab clearance. **a.** Representative images of fluorescent beads (green) uptake in Iba1 (red) positive WT and PPARαKO primary microglia cultures treated with vehicle or 10 μM KDS-5104 for 8 hours. **b.**Quantification of the percentage of microglia positive for the fluorescent beads. **c.** Representative images of CD36 (green) and Iba1 (red) staining in primary WT and PPARαKO microglia cultures treated with vehicle or KDS-5104. **d.** Quantification of CD36 fluorescence intensity per cell. 100 cells per group were analyzed. **e.** Time course of fluorescent labeled Aβ (green) and DAPI (white) in primary WT microglia with or without pretreatment with the CD36 neutralizing antibody followed by vehicle or KDS-5104 treatment. **f.** Analysis of Aβ fluorescence intensity per cell at time of zero of Ab removal. **g.** Quantification of the percentage of baseline fluorescence remaining 1 hour, 2 hour, and 4 hours after the removal of Aβ. Scale bars: 100 μm. For all panels, data are presented as mean ± SEM. Ns: non-significant, *p < 0.05, **p < 0.01, ***p < 0.001, ****p < 0.0001. One way ANOVA with Tukey’s multiple comparisons tests as the post hoc analysis. Each experiment was repeated 2-3 times each in triplicates.

One of the main functions of microglia is to mediate phagocytosis of extracellular materials and clearance by the lysosome. The above experiments established a role of KDS-5104 in regulating lysosomal activity. We next evaluated its effect in phagocytosis. Treating the primary microglial cultures with KDS-5104 resulted in increases of multiple phagocytosis related genes including *Cd68, Fcer1g, Fcgr2b, Trem2, Cd36* (Supplementary Fig. 3a). Supporting a functional role of the phagocytic gene expression, analysis of fluorescently labelled bead uptake showed that KDS-5104 treated WT microglia displayed a higher internalization of beads, but this effect was abolished when PPARα is inactivated (Fig. 3a, b). Among the phagocytic markers that were upregulated by KDS-5104, CD36 is known to be an Aβ scavenger receptor and a downstream gene activated by PPARα^31,32^. Consistent with the RNA expression, immunofluorescence staining showed a PPARα dependent increase in CD36 protein expression in KDS-5104 treated microglia (Fig. 3c, d), which is associated with Increased Aβ engulfment of FITC labelled Aβ (Fig. 3e, f, Vehicle vs. KDS-5104 at 0 hr). In agreement with a functional role of CD36 in Aβ phagocytosis, increased Aβ uptake by KDS-5104 was blocked by pretreating the cultures with a CD36 neutralizing antibody (Fig. 3e, f, CD36 Ab Vehicle vs. CD36 Aβ KDS-5104 at 0 hr). Analysis of Aβ degradation by measuring the percentage of Aβ remaining at various time points post Aβ uptake showed that KDS-5104 treated microglia had a significantly reduced percentage of Aβ remaining compared to the vehicle treated microglia (Fig. 3g, Vehicle vs. KDS-5104). CD36 suppressed microglia also displayed a similar decrease after KDS-5104 treatment (Fig. 3g, KDS-5104 and CD36 Ab KDS-5104), suggesting that CD36 mediates Aβ uptake but not degradation. Altogether, the results combined support a model by which PPARα-CD36 signaling regulates Aβ phagocytosis while PPARα-TFEB interaction promotes Aβ lysosomal degradation.

### KDS-5104 decreases LPS-induced inflammation and lipid droplet formation

**Figure 4:**
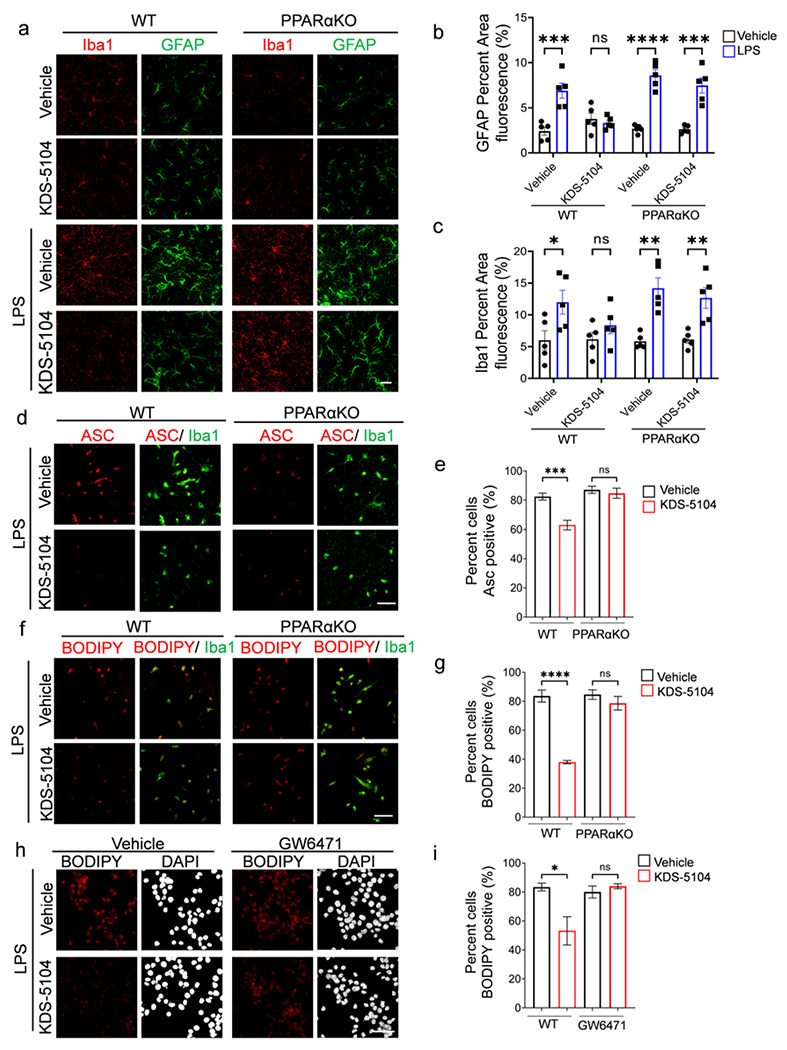
KDS-5104 reduces LPS-induced inflammation and lipid droplet formation via PPARα activation. **a.** Representative images of Iba1 and GFAP from WT and PPARαKO mice pretreated with vehicle or 10 mg/kg KDS-5104 (i.p.) for 24 hours, followed by a co-treatment of LPS (2 mg/kg, i.p.) and KDS-5104 (10 mg/kg, i.p.) for 18 hours *(n=5 mice/group)*. **b.** Quantification of fluorescent area of GFAP in treatment groups. **c.** Quantification of fluorescent area of Iba1 in treatment groups. **d.** Representative images of ASC speck (red) in primary WT and PPARαKO microglia cultures pretreated with vehicle or KDS-5104 for 18 hours before addition of LPS. **e.**Quantification of percentage of ASC positive cells. **f.** Same as (d) except BODIPY+ lipid droplet was imaged. **g.** Quantification of percentage of BODIPY positive cells. **h.** Representative images of lipid droplet formation by BODIPY (red) and DAPI (white) in vehicle or KDS-5104 pretreated and LPS induced BV2 cells without or with GW6471. **i.** Quantification of percentage of BODIPY cells. Scale bar: 100 μm. For all panels, data are presented as mean ± SEM. ns: non-significant, *p < 0.05, **p < 0.01, ***p < 0.001, ****p < 0.0001. One way ANOVA with Tukey’s multiple comparisons tests as the post hoc analysis. The in vitro experiments were repeated 2-3 times each in triplicates.

Given the known anti-inflammatory effect of PPARα^33^, we aimed to determine the role of KDS-5104 in LPS-induced neuroinflammation and test its PPARα dependency. PPARαKO and WT mice received a pretreatment of KDS-5104 (10 mg/kg) for 24 hours, followed by a co-treatment of lipopolysaccharide (LPS; 2 mg/kg) and KDS-5104 (10 mg/kg) for 18 hours. Analysis of Iba1 and GFAP immunoreactivities found similar increases in WT and PPARαKO mice upon LPS treatment (Fig. 4a). KDS-5104 attenuated this effect in WT mice, however, not in LPS treated PPARαKO mice (Fig. 4a-c). LPS also induced ASC specks, an indicator of inflammasome activation, in both WT and PPARαKO microglia cultures (Fig. 4d). KDS-5104 treatment resulted in reduced ASC specks in WT cultures. This suppression was attenuated in PPARαKO microglia (Fig. 4d, e).

Besides the regulation of inflammatory processes, PPARα plays a potent role in fatty acid oxidation and lipid homeostasis^34^. We thus assessed the effect of the KDS-5104-PPARα axis on LPS-induced lipid droplet accumulation. We found that KDS-5104 treatment effectively reduced lipid droplets induced by LPS in WT, but not in PPARαKO microglial cultures (Fig. 4f, g). In support of the genetic knockout, PPARα antagonist GW6471 also blunted KDS-5104 effects on lipid droplet formation in BV2 cells (Fig. 4h, i). Together, these results establish the beneficial effect of KDS-5104 in suppression of inflammation and lipid droplet accumulation and these effects are PPARα dependent.

### KDS-5104 treatment restores lipid dysregulation in 5xFAD mice

Emerging evidence suggests that lipid dysregulation is a key event in the development of AD^35^. Specifically, lipid profile alterations have been identified in microglia with reduced phagocytosis capabilities which can lead to aberrant Aβ accumulation^36,37^. After establishing that KDS-5104-PPARα axis plays a pivotal role in regulating LPS-induced lipid droplet formation, we next aimed to determine the effect of KDS-5104 in AD mouse models.

**Figure 5:**
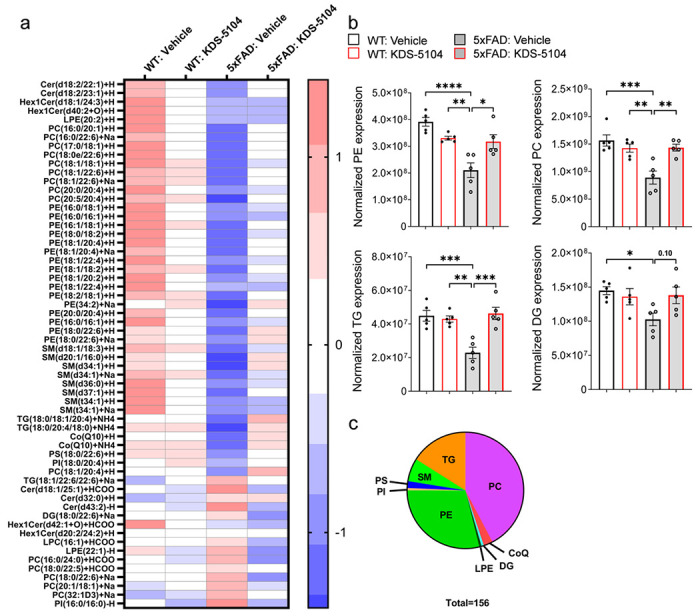
Reversed lipid dysregulation in 5xFAD mice with KDS-5104 treatment. **a.** Heat map representing z scores of top 156 dysregulated lipid species in 5xFAD mice identified by lipidomic analysis of WT and 5xFAD mice treated with vehicle or KDS-5104. **b.** Normalized levels of the lipid species per lipid class; phosphatidylethanolamine (PE), phosphatidylcholine (PC), phosphatidylserine (PS), diglycerides (DG) and triglycerides (TG) per animal *(n=5 mice/group)*. **c.** Pie chart depicts the classes of the 156 lipids identified that are dysregulated in 5xFAD mice compared to WT and rescued with KDS-5104 treatment. For all panels, data are presented as mean ± SEM. Ns: nonsignificant, *p < 0.05, **p< 0.01, ***p< 0.001, ****p< 0.0001. One way ANOVA with Tukey’s multiple comparisons tests as the post hoc analysis.

First, we established a proper dosing to ensure efficacy but no adverse effects, particularly body weight as OEA is known to regulate satiety^5,6^. A subchronic regime was performed where WT mice received a dose of KDS-5104 at either 10 mg/kg or 50 mg/kg every other day for 3 weeks. The 50 mg/kg treatment group had a significant decrease in body weight during the three-week treatment, however, at 10 mg/kg, the body weight was maintained similar to vehicle treatment in both males and females (Supplementary Fig. 4a, b). Regardless, no appreciable differences in animal behavior including rotarod and grip strength were observed in either treatment group (Supplementary Fig. 4c, d). qPCR analysis of the cortex (Supplementary Fig. 4e, f) and the liver (Supplementary Fig. 4g, h) showed increased expression of PPARα and TFEB activities in both tissues demonstrating that 10 mg/kg is a sufficient dosage to achieve in vivo effect. We thus treated WT and 5xFAD mice with KDS-5104 (10 mg/kg, i.p.) or vehicle every other day starting at 2 months of age for a total of 2 months. Untargeted lipidomic analysis of cortical tissue from vehicle or KDS-5104 treated WT and 5xFAD identified a total of 939 distinct lipid species. We generated a heatmap of the z scores of the top 60 lipid species with the most significant changes (Fig. 5a). We further calculated the total abundance of major lipid classes: phosphatidylethanolamine (PE), phosphatidycholine (PC), ceramide (SM), diglycerides (DG) and triglycerides (TG). All except SM showed significant reductions in vehicle treated 5xFAD mice compared to WT controls (Fig. 5b). KDS-5104 treatment resulted in significant increases of PE, PC and TG levels and trended upwards in DG (Fig. 5b). In total, we identified 156 lipids that were dysregulated in vehicle treated 5xFAD compared to WT that was recovered in the KDS-5104 treated 5xFAD (Fig. 5c, Supplementary Table 1).

### Attenuation of Aβ pathology and cognitive deficit by KDS-5104 treatment

**Figure 6:**
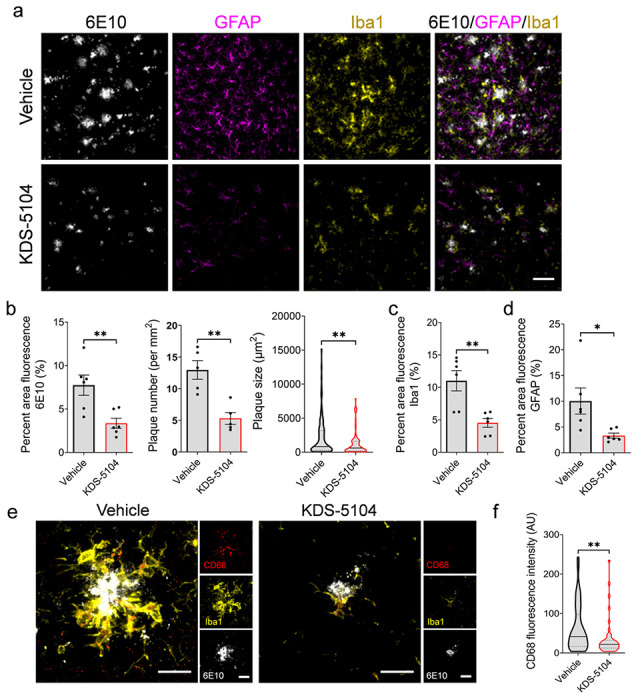
Reduced gliosis and Aβ pathology in 5xFAD mice treated with KDS-5104. **a.** Representative images of 6E10 (white), GFAP (magenta) and Iba1 (yellow) co-staining from the hippocampal sections of 4-month-old 5xFAD mice treated with vehicle or 10 mg/kg KDS-5104 for two months. Scale bar: 100 um. **b.** Quantification of 6E10 positive area, plaque number and size. **c and d.** Quantification of Iba1 (c) and GFAP (d) area fluorescence. **e.**Representative images of CD68 (red), Iba1 (yellow) and 6E10 (white) costaining. **f.** Quantification of CD68 fluorescent intensity. AU: artificial unit. Scale bar: 10 um. For all panels, data are presented as mean ± SEM. *p< 0.05, **p< 0.01 by 2-sided *t*tests.

We next examined the effect of KDS-5104 on Aβ pathology and neuronal function in 5xFAD mice. We used the human Aβ antibody 6E10 to stain brain sections of 4-month-old 5xFAD mice treated with KDS-5104 or vehicle and analyzed the plaque number and size in the hippocampus (Fig. 6a-d). We observed a reduced plaque size and number in 5xFAD mice treated with KDS-5104 compared to vehicle (Fig. 6b). Since PPARα has been implicated in APP metabolism^9^, we examined levels of full-length APP and its C-terminal fragment (CTF) and observed by no appreciable differences between KDS-5104 and vehicle treated 5xFAD (Supplementary Fig. 5). Thus, this reduction is not due to changes in APP expression or processing. Immunofluorescence staining of astrocyte (GFAP) and microglia (Iba1) markers showed that reduced Aβ pathology was accompanied by decreased astrogliosis and microgliosis in 5xFAD mice treated with KDS-5104 (Fig. 6, a, c, d). Characterization of the phagocytic microglia marker (CD68) also found a reduction in KDS-5104 treated 5xFAD mice (Fig. 6, e, f).

**Figure 7:**
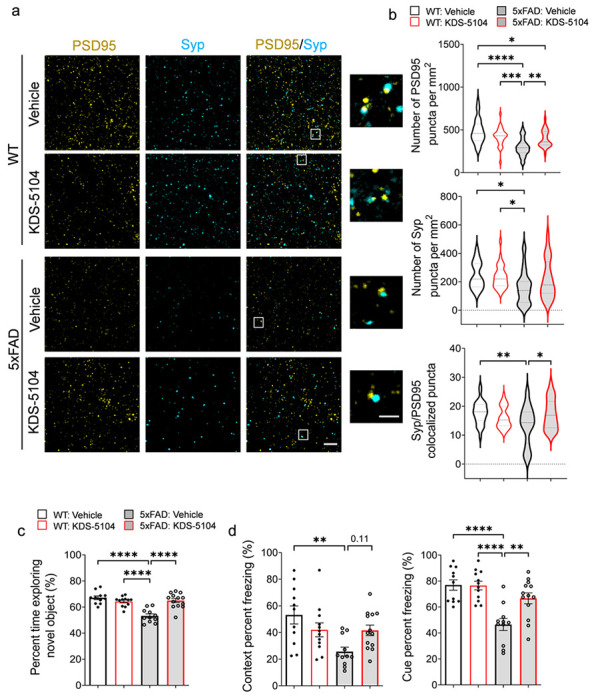
Improved cognitive performance in 5xFAD mice treated with KDS-5104. **a.** Representative images of co-staining of post-synaptic marker PSD95 and presynaptic marker synaptophysin (Syp) from hippocampal CA1 area of WT and 5xFAD mice treated with vehicle or 10 mg/kg KDS-5104. Inset showing enlarged view of Syp and PSD95 co-localized puncta. **b.** Quantification of the number of PSD95, Syp and co-localized Syp and PSD95 puncta *(n=6 mice/group)*. **c.** Quantification of percent time exploring the novel object in NOR assay. **d.** Freezing percentage due to contextual or cue testing in the fear conditioning paradigm *(n=8-10 mice/group)*. Scale bar: 100 um; 25 um in zoomed out image. For all panels, data are presented as mean ± SEM. *p< 0.05, **p< 0.01, ***p< 0.001, ****p< 0.0001. One way ANOVA with Tukey’s multiple comparisons tests as the post hoc analysis.

Having established a reduction in Aβ pathology and gliosis, we next assessed the synaptic and behavioral phenotypes in 5xFAD following KDS-5104 treatment. High resolution imaging of the presynaptic protein, synaptophysin (Syp), and the postsynaptic protein, PSD95, revealed that the overall levels of Syp and PSD95 and colocalization of the pre- and post- synaptic puncta were significantly lower in 5xFAD mice compared to WT controls (Fig. 7a, b). Treatment with KDS-5104 led to increased pre- and post-synaptic markers as well as colocalized synaptic puncta. We further performed cognitive testing to evaluate the functional effect of KDS-5104 treatment. General neurological assessment revealed no group differences in rotarod or grip strength (Supplementary Fig. 6a, b), suggesting no changes in general mobility and motor function between the groups and further verifying the safety of the drug treatment regime. Vehicle treated 5xFAD mice exhibited an increase in distance travelled and total movement time in open field arena suggesting hyperactivity, phenotypes of which was reduced by KDS-5104 treatment (Supplementary Fig. 6c, d). To assess hippocampal-dependent long-term recognition memory, we performed the novel object recognition test (NOR) by measuring the percentage of exploration time of a novel object following a training of two identical objects (Fig. 7c). The four groups did not exhibit object bias during the training phase (Supplementary Fig. 6e). Vehicle treated 5xFAD mice explored the novel object fifty percent of the time, indicating a lack of memory of the novel object. However, KDS-5104 treatment resulted in an increased exploration time of the novel object comparable to the WT mice (Fig. 7c), indicating restored memory. We further performed the fear conditioning assay to test hippocampal dependent (contextual test) and independent (cued test) associative learning. Vehicle treated 5xFAD mice exhibited a decrease in freezing in both the context and cue test compared to WT mice (Fig. 7d). The 5xFAD mice treated with KDS-5104 exhibited a significant increase in freezing in the cue test and trended upwards in the context test compared to vehicle treated 5xFAD (Fig. 7d). Overall, consistent with increased synaptic marker expression, KDS-5104 treatment resulted in improved cognitive performance in 5xFAD mice.

## Discussion

In the current study, we investigated the role of OEA, an endogenous lipid with pro-longevity properties, in AD pathogenesis. Using its stable functional analog KDS-5104, we provide data to show that OEA/KDS-5104 acts on PPARα to activate CD36 and TFEB, leading to enhanced Aβ phagocytosis and lysosomal clearance, respectively, and suppression of LPS-induced lipid droplet accumulation and inflammasome activation in cultured microglial cells. These are associated with normalization of altered lipid profiles, reduction of reactive gliosis and Aβ pathology and improvement of synaptic density and cognitive function in 5xFAD mice. Mechanistically, we reveal a feedforward regulation of the PPARα-TFEB signaling axis and a novel mTORC1 independent activation of TFEB by OEA/KDS-5104, the latter offers potential to bypass the adverse effects associated with mTORC1-dependent TFEB activators such as rapamycin.

Microglia process two major functions: phagocytosis of extracellular materials followed by intracellular clearance and immune and inflammatory pathway regulation. Our in vitro studies demonstrated that OEA/KDS-5104 influence both processes. While we present evidence that CD36 and TFEB mediates microglial Aβ phagocytosis and clearance downstream of PPARα, these effects should not be limited to Aβ as CD36 is known to be involved in lipid sensing and TFEB is a master regulator of lysosomal function inclusive of lipid clearance. The enhanced lipid trafficking and clearance may lead to the suppression of LPS-induced lipid droplet accumulation and inflammasome activation. Alternatively, the OEA-PPARα pathway could function to directly suppress LPS induced changes, which in turn leads to improved microglia phagocytosis and clearance. Of note, lipid droplet accumulating microglia has been shown to be proinflammatory and phagocytosis deficient^37^, further supporting an intertwined relationship between phagocytosis and inflammation.

Our results are to the most part consistent with a recent report that treating the APP/PS1 mice with PPARα agonists led to increased autophagy, reduced Aβ pathology and reversed behavioral deficits^11^. However, in contrast to reduced reactive gliosis observed in our study, Luo et al. reported higher number of astrocytes and microglia in the vicinity of Aβ plaques. Differences in the nature of the compounds and the targets and cell types they engage, treatment regime, and mouse models could all contribute to the apparent discrepancies. For example, besides PPARα, OEA has been reported to bind to other receptors such as GPR119^38^. As such, PPARα independent mechanisms may mediate the OEA/KDS-5104 effect.

OEA is well-known for its function in suppression of food intake and body weight gain, particularly under high-fat diet conditions. This has been reported to be mediated by both the peripheral sensory fibers and through central dopamine signaling^39^. Although we did not observe an overt body weight difference between vehicle and KDS-5104 treated mice with the dose we administered (10 mg/kg), it is still possible that both mechanisms could contribute to the CNS effect we observed. Our result showing elevated PPARα and TFEB signaling in both the liver and the brain by KDS-5104 treatment is in keeping with this idea. Within the CNS, PPARα has been shown to exert its effect in multiple cell types including neurons, astrocytes and microglia^40^. The specific impairment of the PPARα pathway in microglia of the 5xFAD mice prompted us to focus our studies on microglia. However, it is likely that other cell types may also subject to OEA-PPARα regulation, the combination of which could result in the overall beneficial effect of KDS-5104 in 5xFAD mice, including bulk brain lipid profiles and Aβ associated pathologies. In this regard, a recent paper revealed a role of astrocytic PPARα-TFEB and -LDLR pathway in Aβ uptake and clearance^12^. It is known that LDLR is also expressed in microglia and could potentially mediate PPARα-dependent Aβ update in addition to CD36. Nevertheless, our data that Aβ phagocytosis was blocked when primary microglial cultures were treated with the CD36 neutralizing antibody provide strong support for a functional role of CD36 in this process. A microglia specific PPARα knockout will be helpful to address the cell type effect.

Aging is the greatest risk factor for AD. Thus, agents that improve healthy aging may afford benefit in preventing or delaying AD. We present evidence that OEA may represent such a compound. Its reductions in the plasma and CSF of AD patients provide further disease relevance^4^. OEA augmentation offers several attractive features as a therapeutic strategy: First, it boosts an endogenous lipid signaling pathway; Second, it targets two molecules with therapeutic potentials, PPARα and TFEB, and the latter does not require mTORC1; Lastly, OEA is relatively safe and is being marketed as a nutraceutical. Overall, our study calls for further investigation and development of OEA analogs as potential therapy for aging and AD.

## Methods

### Animals

Mice were housed 3–4 mice per cage in a pathogen free mouse facility with ad libitum access to food and water on a 12 hr light/dark cycle. All experiments included approximately equal ratio of male and female mice. All procedures were performed in accordance with NIH guidelines and approval of the Baylor College of Medicine Institutional Animal Care and Use Committee (IACUC). Two-month-old C57Bl6 mice, obtained from the National Institute for Aging, were used for dosage safety study. Mixed gender 5xFAD mice and wild type (WT) littermates were used for chronic KDS-5104 experiments to study effect on amyloid beta pathology. Mixed gender PPARα knockout (PPARαKO) mice (Jackson Laboratory; Strain #:008154; Bar Harbor, ME) and wild type (WT) littermates were used for acute LPS experiments. A minimum of five mice per group were used for all experiments. Some experiments, such as behavioral assays utilized a higher number of mice per group as specified to account for biological variability.

### Human brain specimens

Postmortem brain tissues from AD patients and non-demented controls were provided by the University of Pennsylvania Center for Neurodegenerative Disease Research (CNDR). Informed consent was obtained from all subjects for the use of their postmortem tissues. The demographic data for the human AD and control can be found in Supplementary Table 2.

### Primary culture preparation

Primary microglia monocultures were prepared from mixed gender PPARαKO and WT pups as previously described (^41,42^). Briefly, cortices were isolated from PPARα and wild type newborn pups (P0-P1) and cut finely in dissection media [Hank’s balanced salt solution (HBSS), 10% mM Hepes, and 1% (v/v) penicillin/streptomycin]. Next, tissue was digested with 2.5% trypsin for 15 minutes at 37°C before the addition of trypsin inhibitor (1 mg/ml) for 1 minute. Tissue was then centrifuged for 5 minutes at 1500 rpm. Next, the pellet was triturated, resuspended in complete media [Dulbecco’s modified Eagle’s medium (DMEM) with 10% fetal bovine serum and 1% (v/v) penicillin/streptomycin] and plated onto poly-d-lysine (PDL)–coated T-75 flasks at 50,000 cells/cm^2^ to produce mixed glial cultures. For microglia monocultures, mixed glia cultures were allowed to grow for one week, then microglia were separated from the mixed glia by shaking the confluent flask at 250 RPM for 2.5 hours twice, 48 hours between the two sessions. Following the final shaking, the flasks were tapped on a table and the floated cells, primarily microglia, were collected and seeded in PDL coated 12-well plate or glass coverslips at a concentration of 50,000 cells/cm^2^. Experimentation on microglia monocultures were performed 24–48 hours post seeding.

### FACS based isolation of microglia from adult mouse brain

FACS sorting of microglia was performed as previously described with minor modifications^43^. Briefly, 9-month-old mice were perfused with PBS, brains extracted and gently minced with sterile razor blades. The tissue was digested in papain (Worthington Biochemical) and DNase (Worthington Biochemical), then titrated 5–6 times by a sterile fire-polished glass Pasteur pipette. Next, ice-cold HBSS+ (HBSS with 2mM EDTA and 0.5%BSA) was added and the suspension was pelleted at 310 g for 5 minutes at 4°C. The pellet was resuspended in 1ml of HBSS+, triturated 5–6 additional times, and centrifuged. After centrifugation, the supernatant was filtered through a 40 μm cell strainer (BD Biosciences) and further centrifuged at 310 g for 5 minutes at 4°C. The resulting pellet was resuspended in 20% 4°C Percoll PLUS (Millipore-Sigma) in 1× PBS and centrifuged at 310 g at 4°C for 20 minutes. The resulting pellet was incubated in 500 ul HBSS + containing 1:100 Mouse BD Fc Block (BD Biosciences). Then with the following antibodies: rat anti–CD45-BV421 (1:500, BD Biosciences), rat anti–CD11b-FITC (1:500, BD Biosciences). Microglia population was gated and sorted based on CD45^mid^ and CD11b^+^ expression. Sorting was performed using BD Biosciences Aria II on the 100 μm nozzle. Cells were sorted into 1.7 ml eppendorf tubes coated with 200 μl HBSS+, followed by lysis of pellets in Qiagen RLT buffer containing 1% β-mercaptoethanol for downstream RNA analysis.

### KDS-5104 treatment

For in vitro experiments, cells were plated 24 hours prior to treatment. KDS-5104 was added to the complete media at a concentration of 10 μM unless otherwise stated. Eight hours after addition of KDS-5104, the cells were fixed or collected for further biochemical analysis. In PPARα antagonist experiments, prior to KDS-5104 treatment, cells were pretreated with 10 μM of the PPARα antagonist GW6471 (Cayman Chemical). Cellular model systems used for KDS-5104 treatment include primary microglia isolated from PPARαKO mice and HeLa stable cell line containing the knockout of the three Tfeb family genes; *Tfeb, Tfe3* and *MitF* (TKO)^23^.

For in vivo dosage determination and safety studies we used 2-month-old WT mice treated every other day for 3 weeks with two doses of KDS-5104 (10 mg/kg and 50 mg/kg, i.p.). Vehicle was 0.1% ethanol in PBS. At the conclusion of the treatment, behavioral assays were performed, and mice were sacrificed. Brains were quickly extracted, fixing half for immunofluorescent staining and flash freezing half for biochemical analysis. Liver tissue was also collected for analysis of peripheral changes. The amyloid beta mouse model, 5xFAD and aged matched WT littermates, were used to perform the amyloid pathology studies. At two months of age, each mouse received one treatment of KDS-5104 (10 mg/kg, i.p.) or vehicle, per day for three days a week. The treatment regime was performed for 8 weeks. At the end of the treatment, behavioral assays were performed, and mice were sacrificed. The brain was quickly extracted and frozen or fixed for biochemical analysis.

### LPS treatment

Primary microglia or BV2 cell cultures were plated in 24 well plates with PDL-coated glass cover slips. Cells were treated with 10 μM of KDS-5104 or vehicle. Following an 8-hour incubation, media was replaced by LPS (5 μg/ml) and KDS-5104 (10 μM) containing media. Eighteen hours following LPS application, cells were fixed in 4% PFA in preparation for immunostaining procedures. For lipid droplet assay, following fixation were stained with BODIPY (Invitrogen) and washed with PBS three times. Coverslips were mounted and imaged using confocal microscope.

In vivo LPS treatment was performed as previously described^44^. Briefly, PPARαKO and WT mice received a pretreatment of KDS-5104 (10 mg/kg, i.p.) or vehicle. 24 hours post KDS-5104 treatment, mice received a co-treatment of KDS-5104 (10 mg/kg, i.p.) and LPS (2 mg/kg, i.p.). 18 hours after the LPS injection, tissue was collected and frozen for further analysis.

### Phagocytosis assay

Phagocytosis assays were performed in primary microglia cultures as previously described^45^. Fluorescent latex beads were prepared in FBS for 1 hour at 37°C at a 1:5 ratio. The beads/FBS mixture was then added to prewarmed complete media (1:1000). Beads containing media were added to cells for 1 hour, then removed and washed thoroughly with PBS. Cells were then fixed in 4% PFA and prepared for immunostaining. Cells were analyzed for percentage of microglia with beads internalization. For Aβ uptake and degradation, microglia were pretreated with CD36 neutralizing antibody (2 μg/ml; Abcam) as previously described^46^. Following blocking of CD36, microglia were treated with KDS-5104 for 8 hours (10 μM). Meanwhile, fluorescently labelled Aβ_42_ (Cayman Chemical) was incubated in complete media for 37°C for 1 hour at a concentration of 500 nM. Treated microglia were then incubated in Aβ containing media for 1 hour at 37°C. Media were removed and cells washed. A portion of the cells were fixed at time point 0 and prepared for imaging. The remaining cells were fed with Aβ free fresh complete media for 1, 2 and 4 hours. The cells were fixed, stained, and imaged on confocal with a 40x objective and the fluorescence of Aβ per cell was analyzed using ImageJ software.

### Western blotting

Brain tissue or cell pellets were lysed and homogenized in RIPA buffer containing protease and phosphatase inhibitors. Homogenates were centrifuged at 10,000 g for 15 minutes at 4°C and supernatant was collected. Bicinchoninic acid analysis (Thermo Fisher Scientific) was used to determine and normalize protein concentrations. Protein separation was performed by electrophoresis using 10–15% SDS-polyacrylamide gels. Following separation, proteins were transferred to a nitrocellulose membrane. Nonspecific binding was blocked by 5% BSA in tris-buffered saline then primary antibodies were incubated overnight at 4°C. Primary antibodies were used at the following concentrations; PPARα (1:1000; Cell Signaling), APP (1:1000; recognizes APP-FL and CTF, Cell Signaling), β-actin (1:10,000; Sigma-Aldrich), pS6k (1:1000; Cell Signaling), total S6k (1:1000, Cell Signaling), pAKT (1:1000, Cell Signaling), total AKT (1:1000, Cell Signaling) and γ tubulin (1:1000, Cell Signaling). After primary antibody incubations, secondary antibodies; IR-680-conjugated goat anti-mouse or goat anti-rabbit (1:10,000; Molecular Probes) and IRDye 800-conjugated donkey anti-rabbit or donkey anti-mouse (1:10,000; LI-COR) were used. LI-COR Odyssey machine (LI-COR) was used to image the membranes. The Western blot bands were quantified using ImageJ software (NIH).

### RNA isolation, reverse transcription and qPCR

Total RNA was isolated from cells, human and mouse brain tissues by lysing in Qiagen RLT buffer with 1% β-mercaptoethanol and processed using the RNeasy Mini kit (Qiagen). Reverse transcription was carried out on the isolated RNA using iScript Reverse Transcription Supermix (Bio-Rad). The qPCR analyses were performed using iTaq Universal SYBR Green master mix (Bio-Rad) on a CFX384 Touch Real-Time PCR Detection System. Genes, 18s and GAPDH were used as housekeeping controls. Relative levels of gene expression were quantified by the Bio-Rad CFX manager. Heatmaps were constructed using GraphPad Prism.

### Immunofluorescence

Cells were fixed in 4% paraformaldehyde (4% PFA) and prepared for immunocytochemistry (ICC). Briefly, blocking buffer (0.2% BSA, 0.5% Triton X-100, and 0.05% Tween 20 in PBS) was used to block nonspecific binding sites for 1 hour at room temperature. Next, cells were incubated with primary antibodies: Iba-1 (1:800 or 1:500; Wako or Novus Biologicals, respectively), CD36 (1:1000; Abcam), Lamp1 (1:500; BD Biosceiences). Following primary antibody incubation, cells were incubated with appropriate secondary antibodies (Alexa Fluor 488, 555, or 647; Invitrogen). The nucleus was then stained with 4’,6-diamidino-2-phenylindole (DAPI). Glass cover slips were then mounted and imaged under a Leica TCS confocal microscope. For lysotracker staining, prior to fixation, cells were incubated in complete media containing lysotracker dye (50 nM; Thermo Fisher Scientific) for 30 minutes at 37°C. Cells were thoroughly washed in PBS and fixed for 15 minutes in 4% PFA. Coverslips were mounted and imaged.

For mouse brain tissue, immunohistochemistry was performed on free floating brain sections. Briefly, mice were perfused with saline, brains quickly extracted and fixed in 4% PFA at 4°C overnight. Brains were then transferred to 30% sucrose for 48 hours and microtome-cut into 30 um thick sections. Free floating sections were incubated with primary antibodies: 6E10 (1:1000; BioLegend), Iba1 (1:800 or 1:500; Wako or Novus Biologicals, respectively), GFAP (1:1000; Sigma), CD68 (1:500; BioLegend), PSD95 (1:200; Millipore), synaptophysin (1:500; Abcam). Next, appropriate secondary antibodies (Alexa Fluor 488, 594, or 647; Invitrogen) were used followed by incubation with DAPI. Three sections (including hippocampus and cortex) per mouse brain with five to seven mice per group were used.

### Image quantification

#### Synaptic co-localization

Synaptic marker co-localization analysis was performed with the Imaris software (Oxford Instruments) as described previously^42,44^. Briefly, synaptophysin (Abcam) and PSD95 (Millipore), respective markers for pre- and post-synaptic terminals, were stained in mouse brain sections as described above. Sections were imaged with a 63X oil objective with a 4.0 digital zoom on a Leica confocal microscope. 5 μm thick Z stacks with 0.2 um step size were obtained. Using the Imaris’ ‘Spots’ feature, puncta from each channel were analyzed by generation of spot representation (automatic generation with consistent manual adjustment for all images for accuracy). Total number of spots for each channel were recorded. Spots were then analyzed using the ‘Co-localize Spots’ MATLAB plugin, defining co-localization if the center of the pre- and post-synaptic puncta were within 200 nm.

#### Lysosome characterization

For analysis of lamp1 and lysotracker images (obtained with 63x objective, 2x digital zoom) the Cells feature of Imaris was used. Cell borders and nucleus were defined followed by the generation of representative spots for each marked lysosome (automatic generation with consistent manual adjustment for all images for accuracy). Spots were then analyzed for size, fluorescent intensity, proximity to nucleus and number per cell. Approximately 25 cells per group (obtained from a minimum of 3 separate treatment wells) were analyzed.

### Lipidomics

Brain tissue from 4-month-old WT-vehicle, WT-KDS-5104, 5xFAD-vehicle and 5xFAD-KDS-5104 mice were weighed and normalized. Tissue was then ground using a bead beater. 10 mg of each sample was collected and homogenized in 50 mM Ammonium acetate solution. 10 μl of splash lipidomix Mass Spec Standard (Avanti, 330707) was added to each sample. Following standard addition, lipids were extracted using methanol, metyl terrt-Butyl Ether (MTBE) and water. The samples were then dried in a vacufuge and resuspended in 110 μl isopropanol and methanol (50:50, vol/vol). The samples were analyzed using a Vanquish UPLC and a Lumos orbitrap mass spectrometer (Thermo Fisher Scientific). Analysis of lipidomic data were performed using Lipidsearch software (Thermo Fisher Scientific). The statistical analysis was done by MetaboAnalyst 5.0.

### Behavioral Assays

At the conclusion of animal treatments with vehicle or KDS-5104, mice underwent behavioral testing.Prior to each assay, mice were habituated to the test room for 30 minutes.

#### Open field Arena

Each mouse was placed singly in the center of the open field arena apparatus (OmniTech Electronics, Columbus, OH). Mice were allowed to freely move around the apparatus for 30 min while locomotion activity was recorded using the Versamax activity monitoring software (AccuScan Instruments, Columbus, OH).

##### Novel object recognition:

The novel object recognition protocol included three phases: habituation, a training, and object recognition. All three phase are performed in a Plexiglass arena (measuring 22 cm by 44 cm). The habitation phase included one 5-minute session, in which the animals were allowed to freely explore the arena. Twenty-four hours post habituation, the animals underwent training, in which the mice were placed in the arena with two identical objects. The animals were allowed to freely explore the objects for 5 min. One day after the training phase, the mice underwent testing, in which the mice were placed in the same arena with one object previously explored in the training phase, the familiar object, and one novel object differing in color and shape but sharing a common size and volume. The animals were allowed to freely explore the objects for 5 min. The time spend exploring each object was measured by the ANY-maze software. Exploration of an object was defined by head orientation directed toward the object or physical contact with the object. The object discrimination ratio (ODR) was calculated by the following formula: ODR = (time exploring specified object) / (time exploring novel object + time exploring familiar object) × 100.

#### Fear Conditioning

The fear conditioning protocol involved a training phase, context test, and a cued test. During the training phase, the mice were placed in the conditioning chamber and allowed to freely explore. At 3 min, an auditory stimulus was presented for 30 mins (80-dB white noise) followed by the administration of a foot shock (0.8 mA, 2 s). This was repeated a second time at the 5-min mark. Following training, the mice were then returned to their original housing cages for 24 hours before performing context and cue testing. For the context test, each mouse was returned to the same chamber (same geometric shape of chamber, lights, scents, and auditory sounds) for 5 minutes with no stimulation, freezing was recorded. The cue test was then performed, one hour after the context test. For the cue test, mice were placed in an altered chamber consisting of a different geometric shape, flooring, light brightness, and scent compared to the previous chamber used for training. After 3 minutes, the auditory stimulus was presented for 3 minutes. The software FreezeFrame3 and FreezeView (San Diego Instruments) was used to record and analyze the percent freezing in each trial.

#### Grip Strength

Each mouse was held by the tail near the grid bar of a digital grip strength meter (Columbus Instruments, Columbus, OH) and allowed to fully clasp the grid with both forepaws. The mouse was then pulled parallel away from the meter with a constant force until both forepaws released. The grip strength, measured in kg of force was recorded. The procedure was performed for a total of three trials, averaging the three forces for the final result per mouse.

#### Rotarod

Coordination and motor function were measured by using an accelerated rotating rod test (type 7650; Ugo Basile, Milan, Italy). Mice were placed on the rod (3 cm diameter, 30 cm long) for four trials, with each trial lasting 5 minutes. The rod accelerated from 4 to 40 rpm in 5 min. Latency to fall for each mouse was recorded.

### Statistical analysis

All data were analyzed with GraphPad Prism version 6 and presented as means ± SEM (*p < 0.05, **p < 0.01, ***p < 0.001, and ****p < 0.0001). For simple comparisons, Student’s *t*-tests was used. For multiple comparisons, analysis of variance (ANOVA) followed by Tukey’s multiple comparisons tests as the post hoc analysis were performed. All samples or animals were included in the statistical analysis unless otherwise specified.
